# A Novel Homozygous Germline Mutation in Transferrin Receptor 1 (TfR1) Leads to Combined Immunodeficiency and Provides New Insights into Iron-Immunity Axis

**DOI:** 10.1007/s10875-024-01658-0

**Published:** 2024-01-25

**Authors:** Ümran Aba, İbrahim Cemal Maslak, Canberk İpşir, Damla Pehlivan, Nicholas I. Warnock, Damon J. Tumes, Gökhan Cildir, Baran Erman

**Affiliations:** 1 Department of Pediatric Immunology, Institute of Child Health, Hacettepe University, Ankara, Türkiye; 2 Can Sucak Research Laboratory for Translational Immunology, Hacettepe University, Ankara, Türkiye; 3 Division of Pediatric Allergy and Immunology, Süleyman Demirel University, Isparta, Türkiye; 4 Centre for Cancer Biology, SA Pathology and the University of South Australia, Adelaide, SA 5000 Australia; 5 Institute of Child Health, Hacettepe University, Ankara, Türkiye; 6 UniSA Bradley Building, North Terrace, Adelaide, SA 5000 Australia; 7 Beytepe Campus of Hacettepe University, HUNITEK Building, Floor 1, 06800 Ankara, Türkiye

**Keywords:** Combined immunodeficiency, CD71, iron, T cells, low-density neutrophils

## Abstract

A homozygous missense mutation in the transferrin receptor 1 (TfR1), also known as CD71, leads to a rare inborn error of immunity (IEI) characterized by the impaired lymphocyte activation and proliferation due to defective iron uptake of cells. However, only one causative mutation (c.58T > C, p.Y20H) in the *TFRC* gene coding for TfR1 has been reported so far. We herein identified a new disease-causing homozygous germline mutation in the *TFRC* gene (c.64C > T, p.R22W) (referred to as TfR1^R22W^ from now on) in a Turkish patient with combined immunodeficiency (CID). TfR1^R22W^ results in impaired TfR1 internalization similar to previously defined TfR1^Y20H^ mutation. We found that TfR1^R22W^ is associated with severely restricted B and T lymphocyte clonal diversity and impaired T cell activation and cytokine production as well as defective mitochondrial oxidative phosphorylation in helper T cells. In addition, circulating NK, Treg, and MAIT cell populations were significantly decreased in the patient. Using whole transcriptome analysis, we found dysregulated immune homeostasis and novel biological processes associated with TfR1^R22W^. We also identified a considerable expansion of circulating low-density neutrophils (LDNs) in patient’s PBMCs. Overall, TfR1^R22W^ mutation expands the current understanding of the IEI associated with TfR1 dysfunction and provides new insights underlying impaired immune function, lymphocyte diversity, and granulocyte homeostasis.

**Supplementary Information:**

The online version contains supplementary material available at 10.1007/s10875-024-01658-0.

## Introduction

Iron plays a crucial role in immunity, serving as a vital element for diverse functions of immune cells [1]. TfR1 is a widely expressed heterodimeric type II membrane protein [2]. It is encoded by the *TFRC* gene and plays a key role in regulating iron metabolism [3]. TfR1 binds to transferrin, a protein that carries ferric iron (Fe^3+^) in the bloodstream and mediates its internalization into cells through a process called receptor-mediated endocytosis regulated via the YTRF motif in the cytoplasmic tail of the receptor [4]. TfR1-mediated iron uptake is essential for maintaining proper iron levels within cells, which is crucial for various biological processes [3].

TfR1 dysfunction was previously reported in more than 20 patients with CID [5]. The same homozygous missense variant (p.Y20H), disrupting the YTRF motif and leading to impaired TfR1 internalization and iron uptake, was identified in all patients. Clinical manifestations of these patients include severe infections during childhood, hypogammaglobulinemia, impaired T and B cell function, intermittent neutropenia, anemia, and thrombocytopenia [5–7]. Mild anemia, dysmyelopoiesis, and mental retardation were also observed in some patients [5–7]. While patients have benefited from IgG replacement therapy (IgGRT) and antibiotic treatments, allogeneic hematopoietic stem cell transplantation (HSCT) has proven curative for the disease [5–7].

Herein, we identified a novel germline homozygous missense mutation in the *TFRC* gene and showed previously uncharacterized immunological features of TfR1 dysfunction in a patient with CID.

## Methods

### Study Participants

The blood samples of the patient and healthy controls were obtained in accordance with the local Ethics Committee of the Hacettepe University. Informed consent forms were provided by the participants.

### Mutation Analysis

Genomic DNA was extracted from the peripheral blood using DNA isolation kit (GeneAll). The NGS exome library was prepared using Nextera DNA Prep with Enrichment Kit (Illumina). One hundred fifty base pair paired-end sequencing was performed using the Illumina NextSeq 550 platform. The mapping, variant calling, and annotation were analyzed with SEQ Platform v8 (Genomize). To validate the identified *TFRC* variant, Sanger sequencing was performed in accordance with the standard protocols [8]. Primers used for PCR amplification and Sanger sequencing are listed in Table S1. The amplified DNA region was subjected to sequencing using ABI 3130xl Genetic Analyzer (Applied Biosystems).

### Cell Culture

PBMCs were isolated from the patient and healthy controls using Ficoll-Paque (Capricorn Scientific). Cells were resuspended in RPMI-1640 containing 15% FCS, 10 mM HEPES (Sigma), 100 U/ml penicillin (Sigma), and 200 mM L-glutamine (Sigma). For proliferation assays, cells were stimulated with CD3/CD28 Dynabeads (1:1 ratio, ThermoFisher) or 50 ng/ml PMA (Sigma) and 1 µg/ml ionomycin (Sigma) for 3 days or 200 ng/ml CD40L + 100 ng/ml IL-4 (BioLegend) for 6 days. 0.5 μg/ml or 5 μg /ml ferric ammonium citrate (Sigma) was added into the wells. Proliferation was measured by labeling the cells with 5 μM CFSE (BioLegend) according to the manufacturer’s instructions. For activation assays, cells were stimulated with CD3/CD28 Dynabeads for 24 and 48 h for CD69, CD25, and ICOS expression on 7-AAD − live T cells. For apoptosis assay, PBMCs were stimulated with anti-CD3 (1 μg/ml) and anti-CD28 (2 μg/ml) for 72 h. Apoptotic cells were determined by flow cytometry using FITC Annexin V Apoptosis Detection Kit with 7-AAD (BioLegend).

### Flow Cytometry

Standard flow cytometric methods were used for staining of cell-surface proteins. For intracellular staining, cells were stained with surface markers, fixed, and permeabilized with BD Cytofix/Cytoperm Kit (BD Biosciences) or True Nuclear TF Buffer Set (BioLegend). Flow cytometric tests were performed on FACSCelesta (BD Biosciences). Data were analyzed with FACSDiva (BD Biosciences) and FlowJo software (BD Biosciences). Gating strategy and antibodies used for staining are provided in Supplementary Material.

### In Vitro T Cell Polarization

Naive CD4^+^ T cells were enriched from PBMCs by negative magnetic separation using MojoSort Human CD4 Naive T cell isolation Kit (BioLegend). The purity of isolated cells was checked by flow cytometry (> 95%). 2.5 × 10^5^ naive cells were cultured in flat-bottom 96-well plates with CD3/CD28 Dynabeads in RPMI 1640 supplemented with 15% FCS, 10 mM HEPES (Sigma), 100 U/ml penicillin (Sigma), and 200 mM L-glutamine (Sigma) for 4 days under the following conditions: Th0 (anti-IL-4 (5 μg/ml), anti-IFNγ (5 μg/ml)); Th1 (anti-IL-4 (5 μg/ml), IL-12 (10 ng/ml)); Th2 (IL-4 (10 ng/ml), anti-IFNγ (5 μg/ml)); Th17 (IL-6 (20 ng/ml), TGFβ (1 ng/ml), anti-IFNγ (5 μg/ml), anti-IL-4 (5 μg/ml)) (all recombinant proteins and antibodies were from BioLegend). On day 4, cells were stimulated with PMA (50 ng/ml) and ionomycin (375 ng/ml) for 5 h. Golgi Plug was added into the culture (1:1000 dilution) 1 h after stimulation. The levels of IFNγ, IL-4, and IL-17A were measured by flow cytometry with intracellular staining protocol.

### TfR1 (CD71) Internalization in PHA-Stimulated T Cells

PBMCs from the patient and controls were stimulated with (phytohemagglutinin) PHA for 3 h to increase the TfR1 surface expression in T cells. Then, cells were washed with cold PBS and incubated with mouse anti-human CD71 (5 μg/ml; CY1G4) or mouse IgG2a, κ (BioLegend) for 30 min on ice. Cells were then washed with and resuspended in cold PBS. Baseline CD71 expression was measured using FITC mouse IgG2a, κ (BioLegend). Unstained cells were incubated at 37 °C for 15 and 30 min for CD71 internalization. Endocytosis was ended by incubating the cells on ice and washing with cold PBS followed by surface staining with FITC mouse IgG2a, κ.

### Immune Repertoire Sequencing

RNA was isolated from PBMCs using NucleoSpin RNA Plus Kit (Macherey-Nagel). T cell and B cell receptor (TRA, TRB, and BCRH) libraries were prepared using 100 ng RNA by using SMARTer Human TCR a/b and BCR IgG H/K/L Profiling Kit (Takara) according to the manufacturer’s instructions. The library validation was performed by amplicon size measurement on 2100 Bioanalyzer using the DNA 1000 kit (Agilent). The repertoire library was sequenced on NextSeq 550 platform. The immune profiling data was generated by using Cogent NGS Immune Profiler Software (Takara). Data analyses were performed on R Studio using Immunarch Package (https://immunarch.com/ , https://github.com/immunomind/immunarch) and IMGT (the international ImMunoGeneTics information system) HighV-QUEST and StatClonotype [9].

### Cytokine and Immunoglobulin Measurement

5 × 10^5^ PBMCs were cultured with CD3/CD28 Dynabeads in 96-well plates for 3 days for cytokine production and 5 days for Ig production (200 ng/ml CD40L and 100 ng/ml IL-4 for Ig production). On the last day of culture, Brefeldin A (BioLegend) was added into the culture 4 h before collection of cells. Cultured cells were centrifugated and the supernatant was preserved for bead-based cytokine quantification. Collected cells were used for intracellular staining of IFNγ, IL-4, IL-17A, and IgE. Levels of IL-2, IL-4, IL-10, IL-13, IL-17A, IFN-γ, IgG, IgE, and IgA were quantified using LEGENDplex™ Human Th Cytokine and Human Immunoglobulin Isotype Panels (BioLegend). The analytes were acquired on FACSCelesta Cell Analyzer (BD Biosciences) and Qognit software was used for data analysis.

### Exogenous Expression of FLAG-TfR1 in HEK293T Cells

C-terminally FLAG-tagged TfR1^WT^, TfR1^R22W^, and TfR1^Y20H^ were cloned into pGenLenti vector and verified by Sanger sequencing. One microgram plasmids were transfected into 3 × 10^5^ HEK293T cells using Lipofectamine 3000 reagent in 6-well plates. Forty-eight hours post-transfection, the surface localization of FLAG was analyzed using PE-conjugated DYKDDDDK Tag antibody (CST, #98533, 1:100 dilution) by flow cytometry. From the same samples, total proteins were also extracted using TOTEX buffer. FLAG-TfR1 was detected using mouse anti-FLAG antibody (Sigma BioM2, 1:2000 dilution). β-Actin (CST, #4970, 1:3000 dilution) was used as a loading control.

### Western Blotting

For western blotting, cell lysates were obtained from healthy controls and patient PBMCs using xTractor Buffer (Takara). Proteins were quantified using DC Protein Assay (Bio-Rad) and 20 µg of total protein was loaded on 4–12% precast polyacrylamide gel (Bio-Rad). After proteins were transferred to nitrocellulose membrane by Semi Trans-Blot System (Bio-Rad), the membrane was blocked with 3% BSA in TBST. Blots were incubated with primary antibodies overnight and then HRP-conjugated anti-rabbit IgG antibody (BioLegend) for 1 h. Anti-human beta-actin (Elabscience) was used as a loading control. Blots were observed by ChemiDoc Imaging System (Bio-Rad). Band intensities were measured by ImageLab software (Bio-Rad).

### qRT-PCR

Total RNA was isolated from the PBMCs of patient and healthy controls by using NucleoSpin RNA Plus Kit (Macherey-Nagel). cDNA was generated via iScript cDNA synthesis kit (Bio-Rad). Quantitative real-time PCR was performed with iTaq Universal SYBR Green Supermix (Bio-Rad) in CFX Connect System (Bio-Rad). The primers used for qPCR are shown in Supplementary Table 1.

### Transcriptome Analysis

RNA-Seq libraries were prepared using the Illumina Stranded mRNA preparation kit. Sequencing was performed on an Illumina NextSeq sequencer, producing approximately 36 million, 150-bp paired-end reads per sample. The details of the transcriptome analysis are given in Supplementary Methods.

RNA-Seq data was submitted to NCBI Gene Expression Omnibus (Geo) with the reference number “GSE243237.”

### Metabolic Flux Analysis

Isolated CD4 + T cells were cultured with CD3/CD28 Dynabeads for 24 h. Then, 250,000 cells in Seahorse XF RPMI medium were loaded onto a Seahorse XFp cell culture microplate precoated with poly-L-lysine. The medium was supplemented with 1 mM pyruvate, 2 mM glutamine, and 10 mM glucose for Mito Stress Test. Oxygen consumption rates (OCR) were measured using the Seahorse XFp analyzer (Agilent). Cells were stimulated with additional CD3/CD28 Dynabeads after the equilibration step. Two micromolar oligomycin (18 min), 1 μM FCCP (24 min), and 1 μM rotenone and antimycin A (18 min) were used for injections 1, 2, and 3, respectively. Spare respiratory capacity was calculated as the maximal possible OCR to the basal OCR.

### Statistical Analysis

Statistical analysis was performed by using GraphPad Prism software. One-way ANOVA, Student’s *T*-test, or Tukey’s test was used for the comparisons between the data of the patient and healthy controls. For repertoire analysis, the Wilcoxon rank sum test and the Kruskal-Wallis test were used for the comparison.

## Results

### A Deleterious Missense TFRC Variant in a Patient with Combined Immunodeficiency

The patient is an 18-year-old boy born to first-degree cousins of Turkish origin. His first admission to the hospital was due to unexplained leg bruising at the age of 6. After the detection of thrombocytopenia, he was referred to Hematology Unit. Malignity was not detected in the bone marrow aspiration, but he was diagnosed with idiopathic thrombocytopenic purpura (ITP) and systemic steroid therapy was initiated. After 6-month treatment, there was no recovery in thrombocytopenia. Recurrent sinopulmonary infections, chronic diarrhea, bronchiectasis, microcytic anemia, chronic recurrent neutropenia, and thrombocytopenia were presented since childhood. He also had mild mental retardation, facial dysmorphic features, failure to thrive, and cervical kyphosis (Fig. S2A). Brain MRI did not show any abnormalities for mental retardation (Fig. S2B). At the age of 17, hypogammaglobulinemia was detected and IVIG therapy was initiated, after which the infection severity and frequency were reduced. He was also hospitalized due to pneumonia caused by *Pseudomonas aeruginosa* and received combined treatment with amikacin and meropenem.

**Fig. 1:**
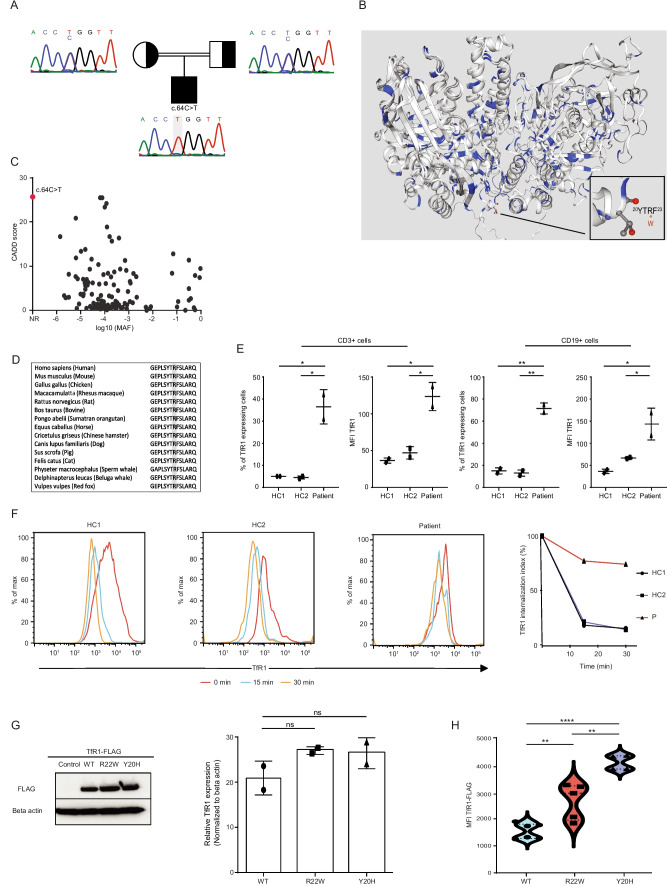
A novel *TFRC* variant and its impact on TfR1 function. **A** The pedigree of the family and the Sanger chromatograms. **B** 3D homology model of TfR1 and the mutant YTRF motif (R-W conversion was indicated) made by SWISS-MODEL. Aromatic amino acid residues were highlighted in blue. **C** CADD vs MAF plot of all homozygous *TFRC* gene variants obtained from the Genome Aggregation Database (gnomAD) v4 datasets. **D** Evolutionary conservation of the TfR1:p.Arg22 residue in different species. **E** TfR1 expression on patient’s and healthy controls’ T and B lymphocytes from 2 independent experiments (flow cytometry). **F** Impaired TfR1 internalization in patient’s PHA-stimulated T cells compared to healthy controls. The percentage of internalized TfR1 was calculated as follows: (MFI at 0 min − MFI at specified time point) × 100/MFI at 0 min. **G**,** H** FLAG-tagged TfR1 expression in transfected HEK293T cells as determined by western blotting in total lysates (**G**) and flow cytometry on the cell surface. Western data is representative of 2 independent experiments. Beta actin was used as loading control. MFI mean fluorescence intensity, HC healthy control, MAF minor allele frequency, NR not reported, WT wild type (*0.05, **0.01, and ****0.0001)

In laboratory findings of the patient, low IgA and IgG levels with inverted CD4/CD8 ratio were found (Table 1). WES was performed to identify the underlying genetic defect of a possible IEI, which revealed a novel homozygous missense mutation in the *TFRC* gene, (c.64C > T, p.Arg22Trp) (Table S2), which was also confirmed by Sanger sequencing. Both parents were found to be heterozygous carriers (Fig. 1A). The identified variant is very rare in the population database gnomAD v4 (only 7 heterozygous carriers) and absent in our local database (*n* =  ~ 10k). It was predicted as “probably damaging” and “deleterious” by PolyPhen-2 and SIFT, respectively, and CADD score was 25.7. The variant affects the YTRF motif in the cytoplasmictail of TfR1 (Fig.1B) and has not previously been reported as a homozygous variant in gnomAD v4 (Fig. 1C). Arginine (R) residue in the YTRF motif is evolutionary well-conserved in different species (Fig. 1D).

**Table 1:** Laboratory findings of the patient

Complete blood counts	Results	Reference values [34, 35]
WBC (10^9^/l)	6.4	4.3–11.3
Lymphocyte (%)	**47.2**	20–40
Neutrophil (%)	**19.4**	40–60
Eosinophil (%)	2.9	1–4
Monocyte (%)	**30.1**	2–8
Platelets (10^9^/l)	**81**	155–435
Hemoglobin (g/l)	118	110–150
MCV (fl)	**69**	73.7–95.5
MPV (fl)	11.1	6.3–11.2
PT (%)	**94**	-
Lymphocyte subsets	**%/count**	
CD3^+^ T cells	76	58–82/1.1–4.1
CD4^+^ T cells	29/0.88	26–48/0.6–2.4
Naive	**71**/0.62	13.9–66.4/0.4–2
Memory	28/0.24	8–28/0.2–0.8
Central memory	**6.7/0.06**	21.7–67 (%)
Effector memory	22/0.2	2.9–24.6 (%)
TEMRA	2.4/0.02	**0.3–44.6 (%)**
CD8^+^ T cells	**47**/1.4	16–32/0.4–1.5
Naive	**87**/1.2	4.1–67.5/0.3–1.5
Memory	13/0.18	11.5–72.5/0.05–0.4
Central memory	**0.9**/0.01	1.4–27.9 (%)
Effector memory	13/0.18	0.7–39.9 (%)
TEMRA	**75/1.1**	3.6–66 (%)
CD19^+^ B cells	18/0.5	10–30/0.2–1.4
Naive	**98/0.49**	33.7–79.2/0.05–0.3
Memory	**2/0.01**	5.3–31.6/0.02–0.13
Switch memory	**0.4**	-
Marginal zone	**0.1**	-
CD16^+^CD56^+^ NK cells	**2/0.06**	8–30/0.2–1
Other cell subsets		
CD4 + CD25 + FOXP3 + Treg cells (%)	**1.5**	HC1 6 HC2 7
CD3 + CD4- CD161 + TCR Vα7.2 + MAIT cells (%)	**1**	HC1 8 HC2 7
CD4 + CD45RA-CXCR5 + Tfh cells (%)	8	HC1 9 HC27
CD14-CD16 + CD66b + CD15 + LDN (% in PBMCs)	**19**	HC1 0.2 HC2 0.5
Immunoglobulins		
IgA (mg/dl)	**1**	139–378
IgG (mg/dl)	**76**	913–1884
IgM (mg/dl)	**576**	88–332
IgE (IU/ml)	0.1	**-**
Other tests		
CRP (mg/l)	**111**	0–5
B12 (pg/ml)	**626**	126–505
Ferritin (ng/ml)	**10.4**	24–336
TIBC (µmol/l)	**147.87**	42.96–80.55

*WBC* white blood cell, *MCV* mean corpuscular volume, *MPV* mean platelet volume, *PT* prothrombin time, *Treg* regulatory T cell, *MAIT* mucosal-associated invariant T, *TEMRA* terminally differentiated effector memory, *LDN* low-density neutrophil, *CRP* C-reactive protein, *TIBC* total iron binding capacity. The numbers in the brackets indicate the test units. Bold numbers indicate aberrant values

Detailed immunologic analysis (Table 1) identified an accumulation of CD8 + terminally differentiated effector memory (TEMRA) cells and near absence of memory B cells in the TfR1^R22W^ patient. In addition, low levels of NK, regulatory T (Treg), and mucosal-associated invariant T (MAIT) cells were detected.

### Increased TfR1 Surface Expression and Impaired TfR1 Internalization in the Patient

We observed that TfR1 surface expression is significantly higher in patient’s T and B lymphocytes at steady state (Fig. 1E). To investigate if TfR1^R22W^ patient’s lymphocytes have impaired TfR1 internalization, we performed an assay in PHA-stimulated T cells, which promptly upregulate surface expression of TfR1 upon stimulation. Notably, after incubation with an anti-TfR1 antibody, surface expression of TfR1 was significantly decreased due to receptor internalization in PHA-stimulated CD3^+^ T cells of healthy controls. However, the internalization rate of TfR1 was approximately fourfold lower in TfR1^R22W^ patient’s T cells (Fig. 1F)*.* To prove causality of TfR1^R22W^, we transfected equal amounts of C-terminally FLAG-tagged TfR1^WT^, TfR1^R22W^, and TfR1^Y20H^ expressing plasmids into HEK293T cells and observed that the surface expression of FLAG-tagged TfR1 was significantly higher in cells with mutant residues despite comparable expression in total lysates of cells (Fig. 1G, H). These data show that both Y20 and R22 residues are critical for internalization of TfR1, and their mutation leads to increased residence of TfR1 on the cell surface.

### Impaired Lymphocyte Function in the Patient

**Fig. 2:**
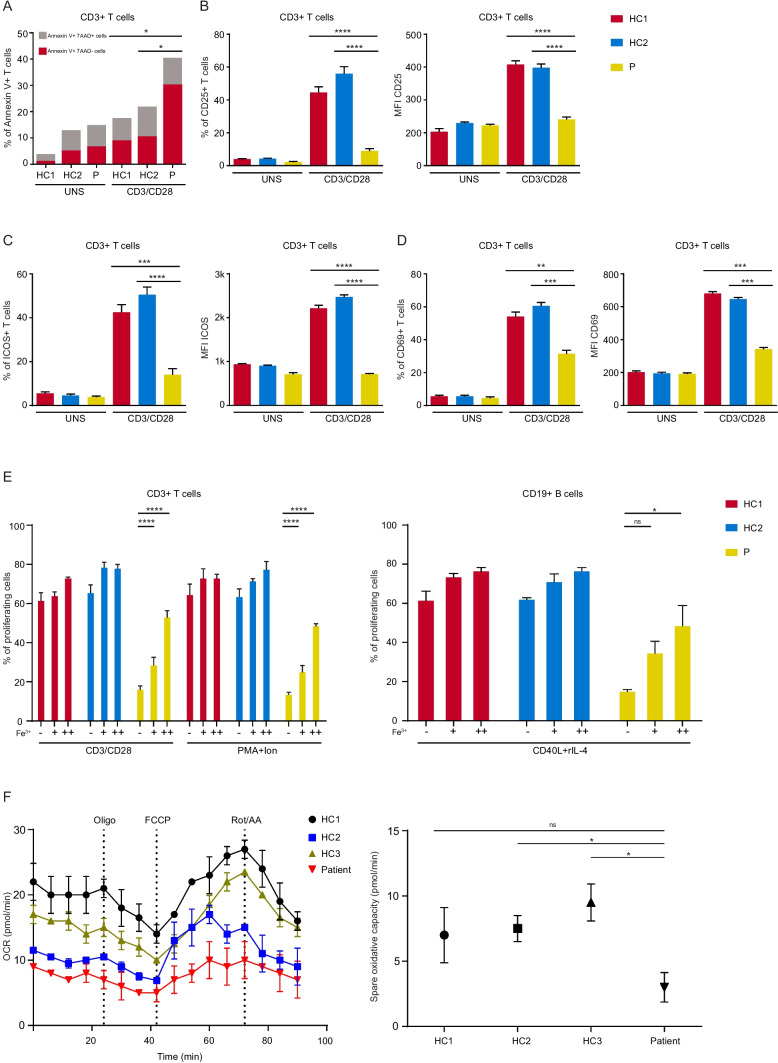
Activation, proliferation and metabolic profiles of the patient’s cells compared to the healthy controls. **A** Flow cytometric analysis of the early apoptotic (Ann V + 7-AAD −) and apoptotic (Ann V + 7-AAD +) T cells from healthy controls and patient. **B**–**D** CD25, ICOS, and CD69 expressions on T lymphocytes on days 1 and 2 after TCR stimulation via CD3/CD28 beads. Results were obtained from 2 independent experiments. **E** Impaired lymphocyte proliferation in the patient, compared to healthy controls and the effect of the extrinsic iron citrate in proliferation. T cells were stimulated by CD3/CD28, PMA, and ionomycin for 3 days, while B cells were stimulated by CD40L and rIL-4 for 5 days. Bars represent means ± SEM of percent of proliferating cells from two independent experiments. **F** Impaired mitochondrial function in the patient, compared to healthy controls. Oxidative capacity was measured by two independent mitochondrial stress tests in TCR-stimulated CD4^+^ T cells. UNS unstimulated, HC healthy control, Fe^3+^  +  = 0.5 µg/ml, +  +  = 5 μg/ml, PMA phorbol 12-myristate 13-acetate, Ion ionomycin, OCR oxidative consumption rate, ns not significant (*0.05 and ****0.0001)

To investigate the effects of iron deficiency in lymphocyte activation, we examined activation markers on T lymphocytes after TCR stimulation. We first checked the effect of TfR1^R22W^ on activation-induced T cell apoptosis. Notably, Annexin V + early apoptotic T cells were significantly increased in patient cells after 72-h TCR stimulation compared to healthy controls (Fig. 2A). Furthermore, patient T cells failed to upregulate the activation markers CD25 and ICOS whereas the early activation marker CD69 was increased nearly as half of the control T cells after TCR-mediated activation (Fig. 2B–D). T cell (CD3^+^) proliferation following TCR or PMA/ionomycin activation and B cell (CD19^+^) proliferation in response to CD40L and rIL-4 were defective in patient cells compared to healthy controls (Fig. 2E). Impaired proliferation was partially rescued with the addition of an independently internalized iron source (ferric ammonium citrate) reinforcing that the proliferation defect was due to impaired iron uptake (Fig. 2E).

Mitochondria is one of the main organelles utilizing the endocytosed iron in the cells. To investigate whether mitochondrial respiration is altered due to TfR1^R22W^ mutation, we performed Seahorse Mito Stress Test and observed significantly impaired oxidative capacity in TfR1^R22W^ CD4 + T cells following TCR stimulation (Fig. 2F).

**Fig. 3:**
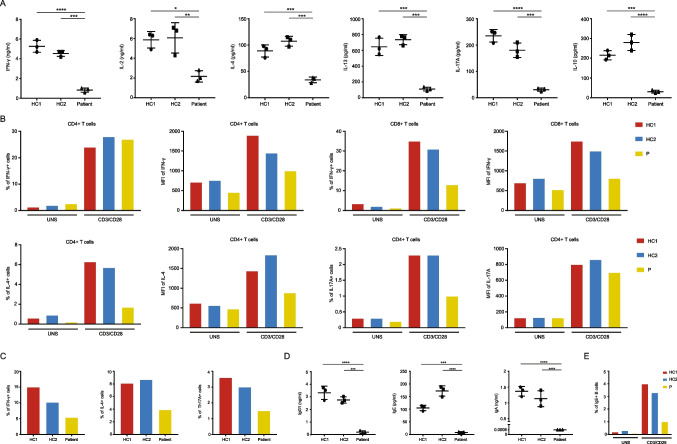
Cytokine and immunoglobulin production and T helper cell polarization profiles in patient’s cells and healthy controls. **A** Cytokine measurements in supernatants of PBMC cultures. Cells were stimulated by CD3/CD28 beads for 3 days. **B** Flow cytometric data of cytokine expressions in T cells. Cells were stimulated by CD3/CD28 beads for 3 days. **C** Th1, Th2, and Th17 polarization of T lymphocytes. Cells were stimulated by polarization conditions for 4 days and additional PMA + Ion for 5 h. Th1, Th2,and Th17 populations were characterized by intracellular measurement of IFNγ, IL-4, and IL17A, respectively. **D** Ig measurements in supernatants of PBMC cultures. Cells were stimulated by CD40L + rIL-4 for 5 days. **E** Flow cytometric data of IgE expression in B cells. Cells were stimulated by CD40L + rIL-4 for 5 days. Cytokine and Ig measurements in culture supernatants obtained from 3 independent experiments. HC healthy control, UNS unstimulated, MFI mean fluorescence intensity (*0.05, **0.01, ***0.001, and ****0.0001)

We found impaired cytokine production in TfR1^R22W^ T cells after TCR stimulation. CD3/CD28 stimulation revealed significantly decreased IFN-γ, IL-2, IL-4, IL-13, IL-10, and IL-17A levels in culture supernatants (Fig. 3A). Moreover, intracellular cytokine staining from the same cultures showed impaired production of IFN-γ, IL-4, and IL-17A (Fig. 3B). We also performed T helper cell polarization assay and observed defective Th1, Th2, and Th17 differentiation in TfR1^R22W^ T cells (Fig. 3C) [10, 11]. We also found decreased IgG, IgE, and IgA production after CD40L and rIL-4 stimulation of patient PBMCs (Fig. 3D, E).

### Restricted Immune Repertoire due to Causative TFRC Variant

**Fig. 4:**
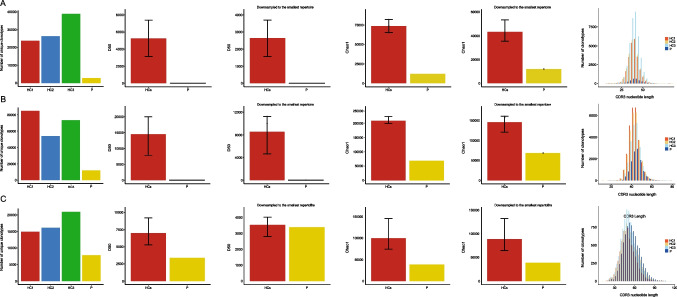
TCR and BCR repertoire profiles. **A** TRA (T cell receptor alpha chain) repertoire. **B** TRB (T cell receptor beta chain) repertoire. **C** BCR M (B cell receptor μ chain) heavy chain repertoire. First graphs show the number of unique clonotypes in T and B cells. Diversity indexes were given as d50 and Chao1. We also downsampled the sequencing data to the smallest repertoire by the clones to prevent any bias due to the differences of the number of total reads. Last graphs show CDR3 nucleotide lengths. Although there are notable differences among the patient and healthy control groups, the Wilcoxon rank sum and the Kruskal-Wallis tests’ results are not significant possibly due to number of the patient, only one. HC healthy control, P patient

RNA sequencing of TCR alpha and beta chains (TRA and TRB) and BCRµ chain (BCRM) revealed restricted repertoire profiles in the patient (Fig. 4A–C). The number of unique clonotypes of all chains in the patient was lower than age-matched control samples. Repertoire diversity analyses of CDR3 regions calculated by d50 and Chao1 indexes showed that TCR diversity was very restricted in comparison to healthy controls. The amount of the most frequent clonotypes which is the other way of the estimation of repertoire diversity revealed that top 100 shared unique TRA and TRB clonotypes occupy approximately 60% of the total repertoire in the patient (Fig. S3A). To predict potential autoreactivity of T cells [12], hydrophobicity of amino acid residues at positions 6 and 7 of CDR3 was assessed. However, there was no accumulation of hydrophobic residues at those positions in patient T cells (Fig. S3B). B cell heavy chain diversity was also restricted in the TfR1^R22W^ patient. However, after normalization of the data to the smallest number of total aligned BCR clones among the samples, one of the diversity indexes, d50, showed comparable result with the healthy controls. Top clonal proportion of top 100 shared unique clonotypes supported this with very low occupation in total BCR repertoire (Fig. S3A). Similar to the repertoire diversity, CDR3 lengths were noticeably shorter in patient’s TCR while BCR CDR3 length was comparable to healthy controls (Fig. 4A–C).

We also sequenced BCR γ chain repertoire regions but there were only a few clones in the patient possibly due to near absence of memory B cells.

### Dysregulated Gene Expressions in the Patient PBMCs

**Fig. 5:**
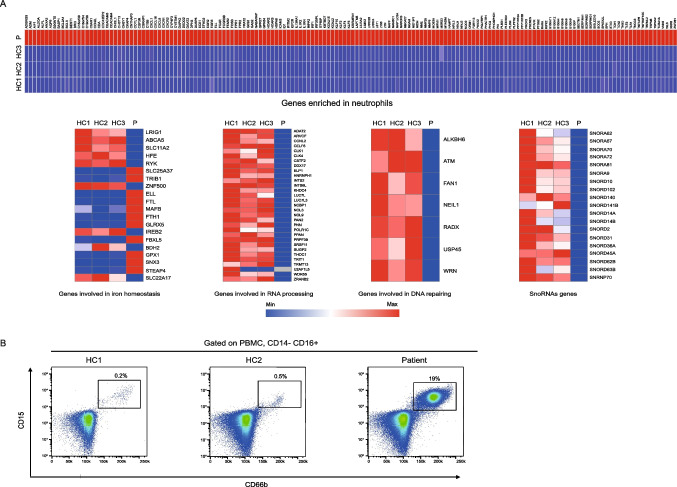
The transcriptomic data from PBMCs. **A** mRNA transcriptome data of PBMCs. Heatmaps show the differentially expressed genes associated with neutrophil functions (> 4-fold difference), iron homeostasis, RNA processing, DNA repairing, and SnoRNA genes. **B** Percentages of low-density neutrophils. HC healthy control, P patient

Analysis of whole transcriptome RNA-Seq data from patient’s PBMCs and three age-matched healthy controls revealed dysregulation of a large number of genes associated with the TfR1^R22W^ variant. When the most highly differentially expressed genes (> 4-fold difference) were investigated, the most notable set of upregulated genes in patient’s PBMCs were identified to be highly or exclusively expressed in neutrophils (Fig. 5A) [13, 14]. Consistent with these results, we identified a greatly expanded population of low-density neutrophils (LDNs) (CD14^−^CD16^+^ CD66b^+^CD15^+^) in patient’s PBMCs using flow cytometry (Table 1 and Fig. 5B). Unlike conventional neutrophils, which are found in the granulocyte fraction of Ficoll-mediated density gradient centrifugation, LDNs are found in the PBMC fraction due to their lower density [15]. Dysregulated genes in patient PBMCs also include genes involved in the regulation of the iron homeostasis (Fig. 5A)*.* While some genes regulating iron metabolism such as ABCA5 and SLC11A2 are downregulated, genes such as FTL, FTH1, and SLC25A37 are upregulated in TfR1^R22W^ patient PBMCs (Fig. 5A). Another notable set of dysregulated genes is involved in “RNA processing and splicing” and “DNA repair” (Fig. 5A) and small nucleolar RNAs (snoRNAs). Several genes were selected and the expression levels were confirmed by qRT-PCR (Fig. S3C). The overall impact of TfR1^R22W^ at the cellular and organismal level is depicted in Fig. S4.

## Discussion

TfR1 deficiency was identified as a cause of combined immunodeficiency with remarkable immune system dysfunctions such as defective lymphocyte proliferation and unresponsiveness to severe infections [5].

There are more than 20 patients with TfR1 deficiency in the literature. All identified patients were from Kuwait and Saudi Arabia with Arabic origin and had the same variant (c.64C > T, p.Tyr20His) in the *TFRC* gene. Patients were characterized with severe clinical presentations including recurrent sinopulmonary infections, hypogammaglobulinemia, chronic diarrhea, failure to thrive, and intermittent cytopenia. Mild anemia, dysmyelopoiesis, and mental retardation might be seen in some patients as well. Our patient has almost all these common symptoms.

In detailed immunologic workup, in addition to hypogammaglobulinemia and aberrant CD4/CD8 ratio, accumulation of CD8 + TEMRA cells and very low memory B cell ratio have been revealed consistent with the previously identified patients with TfR1^Y20H^ mutation [5, 6]. However, the observation of lower levels of Tregs and MAIT cells is novel in this study.

Tyrosine-based sorting motif YXXØ (where X is any amino acid and Ø is a bulky hydrophobic amino acid) is present in the cytosolic tail of many cell surface receptors. In the context of TfR1, this motif is present in the form of YTRF which is affected by the identified TfR1^R22W^ variant in the patient. Several biochemical studies have reported the critical role for tyrosine residue in this motif [16]. However, the role of other residues in YTRF motif in the regulation of TfR1 internalization is not well-studied. Although a study reported that TfR1^F23Y^ mutation, but not TfR1^T21F^ mutation, leads to a significant decrease in the internalization rate of TfR1, the role of R22 residue is less clear [16]. Using peptide library screening, it was observed that arginine (R) in the Y + 2 position (ArgY + 2) of YXXØ motif is preferred over other amino acids suggesting the relative importance of ArgY + 2 (corresponding to TfR1^R22^) [4, 17]. Our clinical observations and cellular internalization assays also support this notion.

The X-ray structure of the ectodomain of TfR1 has been reported in several studies but these structures lack the N-terminal cytoplasmic tail of the receptor to be able to interpret the impact of TfR1^R22W^. However, the structure of the peptide of the internalization motif of trans-Golgi network protein (TGN38) in complex with μ2 is available (PDB: 1BXX) [18]. In this peptide (DYQRLN), tyrosine (Y), and arginine (ArgY + 2) are conserved as in the YTRF motif of TfR1 (ArgY + 2) forms hydrophobic interactions with Ile419 and Trp421 and makes a hydrogen bond with Lys420 of μ2 [4]. Given ArgY + 2 is also conserved in YTRF motif of TfR1, TfR1^R22W^ mutation is expected to disrupt these interactions because tryptophan with an aromatic side chain does not have a hydrogen atom in a position to participate in the same type of hydrogen bonding interaction.

Low cytoplasmic iron is known to increase the surface expression of TfR1 [19]. Accordingly, we observed that TfR1 surface expression is significantly higher in patient’s T and B lymphocytes at steady state. Supporting these notions, exogenous expression of equal amounts of FLAG-tagged TfR1^R22W^ or TfR1^Y20H^ resulted in an increased staining on the surface of HEK293T cells compared to TFRC^WT^ due to impaired shuttling between plasma membrane and endosomes. We also observed that impact of TFRC^Y20H^ on endocytosis was more pronounced than TFRC^R22W^ consistent with the essential role of tyrosine in the YTRF motif.

Iron has been shown to be involved in critical cellular functions in white blood cells, which have lineage- and cell type–specific demands for this trace element. In the context of lymphocytes, although T and B cells do not express TfR1 in the steady state, they rapidly upregulate it on the cell surface upon activation [20, 21], which regulate a multitude of cellular parameters including the activation of different signal transduction pathways, adhesion, metabolic regulation, and proliferation [22, 23]. Accordingly, defective iron uptake upon activation of our patient’s T cells was associated with impaired activation, proliferation, mitochondrial metabolism, and cytokine-induced polarization. The reduction in CD25 and ICOS in TfR1^R22W^ patient’s T cells suggests that late activation leading to proliferation was more significantly impaired due to TfR1 dysfunction [24]. Proven defective lymphocyte proliferation in TfR1^R22W^ patient cells was recovered with the exogenic iron confirming that the proliferation defect was due to iron deficiency. Moreover, increased apoptosis rate may also contribute to the impaired proliferation of TfR1 mutated T cells. Since oxidative phosphorylation in mitochondria depends on iron [11], mitochondrial respiration was significantly affected in the patient. Although there are controversial results regarding cytokine production associated with iron deficiency and repletion [22, 25], we found impaired cytokine production in TfR1^R22W^ T cells after TCR stimulation. Similarly, in vitro B cell class switching is significantly impaired in patient’s cells in agreement with very low immunoglobulin levels in vivo.

Since TfR1-mediated iron uptake is required for lymphocyte development [1], we reasoned that TCR and BCR repertoire might be affected in our patient. Our results showed that TCR repertoire was very restricted due to TfR1 deficiency while BCR heavy chain repertoire was less affected. This is in line with the results from the study by Ned et al. [1] in which they showed that B cell maturation is less dependent on TfR1 pathway.

Dysregulated gene expressions in the patient PBMCs revealed a notable set of upregulated genes associated with neutrophils [14, 26]. In accordance with this observation, we detected very increased population of LDNs in the patient. Iron modulates neutrophil differentiation in a cell intrinsic manner [27]. Consistent with the greater iron demand in neutrophil development, neutrophil progenitors in human bone marrow highly express TfR1, which is progressively downregulated during their development into mature neutrophils [28]. The fact that our patient has intermittent neutropenia is in line with the importance of high cellular iron demand in neutrophil differentiation. In stark contrast to neutrophils, significantly higher levels of monocytes and LDNs in patient’s PBMCs compared to the healthy controls highlight the disruption of neutrophil-monocyte ontogeny due to TfR1^R22W^-mediated dysregulation in iron homeostasis.

We suspect that the balance between conventional neutrophils and LDNs might be disrupted due to dysregulation of iron uptake. This is evident in our patient, who has lower levels of conventional neutrophils and very high levels of LDNs. It is also important to note that an increased LDN population in PBMCs has been reported in several inflammatory diseases, such as sepsis [29] or autoinflammation due to NEMO deficiency [30]. Therefore, distinct or overlapping mechanisms might be responsible for the expansion of LDNs in our patient due to the homozygous TfR1R22W mutation.

Down- or upregulated genes associated with iron metabolism suggest a broader dysregulation of iron homeostasis [31]. Genes involved in “RNA processing and splicing” and “DNA repair” provide an interesting link between iron metabolism and these processes. Indeed, this is in line with a recent study showing dysregulated expression of genes involved in RNA processing, alternative splicing, and DNA repair upon overexpression of TFRC in human renal tubular mesangial cells (HRMCs) [32].

It is important to note that, in addition to the immune system aberrations, neurological problems in the patient (intellectual disability, failure to thrive, and facial dysmorphism) are consistent with the importance of iron-transferrin-TfR1 axis in the nervous system [33].

One limitation of our study is the single-patient nature of our findings. Although we have observed many of the clinical characteristics reported in patients with the TfR1^Y20H^ mutation in our patient, identifying additional patients with the TfR1^R22W^ mutation would strengthen our findings. For instance, our findings of a significantly expanded LDN population were not previously reported in patients with the TfR1^Y20H^ mutation. It remains to be seen whether this is a common mechanism of iron deficiency due to TfR1 dysfunction.

In conclusion, we have identified several phenotypes associated with TfR1 dysfunction due to novel causative mutation in the *TFRC* gene. Our results also provide new insights into critical roles of iron uptake in innate and adaptive immunity.

### Supplementary Information

Below is the link to the electronic supplementary material.

Supplementary file1 (PDF 21695 KB)

## Data Availability

The datasets generated during the RNA-Seq are available in the NCBI Gene Expression Omnibus (Geo) with the reference number “GSE243237.”
