# Supplementary material for: A Novel Homozygous Germline Mutation in Transferrin Receptor 1 (TfR1) Leads to Combined Immunodeficiency and Provides New Insights into Iron-Immunity Axis
Source: J Clin Immunol. 2024 Jan 25;44(2):55. doi: 10.1007/s10875-024-01658-0 (PMC10811203; doi:10.1007/s10875-024-01658-0)
Supplement: Supplementary file 1 — Supplementary file1 (PDF 21695 KB) [file 10875_2024_1658_MOESM1_ESM.pdf]

A novel homozygous germline mutation in transferrin receptor 1 (TfR1) leads to combined immunodeficiency and provides new insights into iron-immunity axis

Journal of Clinical Immunology

Ümran Aba[1,2]*, İbrahim Cemal Maslak[3]*, Canberk İpşir[1,2], Damla Pehlivan[2], Nicholas I. Warnock[4], Damon J. Tumes[4], Gökhan Cildir[4†], Baran Erman[2,5†]

[1] Department of Pediatric Immunology, Institute of Child Health, Hacettepe University, Ankara, Türkiye

[2] Can Sucak Research Laboratory for Translational Immunology, Hacettepe University, Ankara, Türkiye

[3] Division of Pediatric Allergy and Immunology, Süleyman Demirel University, Isparta, Türkiye

[4] Centre for Cancer Biology, SA Pathology and the University of South Australia, Adelaide, SA 5000, Australia

[5] Institute of Child Health, Hacettepe University, Ankara, Türkiye

*These authors contributed equally to this work and are co-first authors

† These authors contributed equally to this work and are co-senior authors

Correspondence:

Gokhan Cildir, UniSA Bradley Building North Terrace GPO Box 2471 Adelaide SA 5001| IPC: CWE-44 Australia, +61 8 830 27922, Gokhan.Cildir@unisa.edu.au

Baran Erman, Beytepe Campus of Hacettepe University HUNITEK Building Floor 1 06800 Ankara Türkiye, +90 312 297 63 61, baranerman@gmail.com

Supplementary methods

Flow cytometry antibodies

For immunophenotyping, activation and proliferation assays, following anti-human monoclonal antibodies and required isotype controls were used: CD3-PE (OKT3), CD3-APC (OKT3), CD3- PB (UCHT-1), CD4-BV510 (SK3), CD8-BV421 (SK1), CD69-PE(FN50), CD185-FITC (J252D4), CD45RO-BV421 (UCHL1), CD8-PERCP (BD, SK1), CD197-PE (G043H7), IL-4-PE (MP4-25D2), CD278-PE (C398.4A), CD25-FITC (M-A251), TCRvα 7.2-AF700 (3C10), CD45RA-APC (HI100), CD161-PB (HP3G10), CD31-PE (WM59), CD56-AF488 (B159), CD56- PE (MY31), CD19-APCCy7 (SJ25C1), CD86-PE (IT.2), FOXP3-PE (206D), CD16-APC (B73.1), IL-17A-FITC (TC11-18H10.1), CD38-APC (HIT2), IgD-BV421 (IA6-2), IgE-AF647 (MHE-18), CD27-APC (M-T271), IgM-FITC (G20-127), IL-21-PE (3A3-N2), CD71-FITC (CY1G4), CD15- PERCP (W6D3), CD14-FITC (M5E2), CD66b-PB (G10F5), IFNγ-CoraLite488 (Proteintech, 1E10G7). Unless otherwise noted, all antibodies were purchased from BioLegend.

Transcriptome analysis

Raw reads were analyzed using FastQC (version v.0.11.0, http://www.bioinformatics.bbsrc.ac.uk/projects/fastqc) to assess quality and adapter contamination. Adapters were trimmed using cutadapt [1] (version 1.18) with the following parameters: -a CTGTCTCTTATACACATCT -A CTGTCTCTTATACACATCT --minimum-length=18 --error- rate 0.2 --overlap=5. Trimmed reads were aligned to the hg38 reference genome using STAR (version 2.7.10a) producing a gene count matrix [2]. Library size normalization factors were calculated in R using the calcNormFactors function from the edgeR (version 3.38.4) Bioconductor package, allowing within gene comparisons between samples [3]. Counts were transformed to counts per million using the cpm function from edgeR.

Supplementary tables

Supplementary Table 1. Primers used for Sanger sequencing and qPCR

Method	Gene	Forward sequence	Reverse Sequence
**PCR and Sanger**	*TFRC*	CCCGTGCCTGTTCTTCATCA	AGCTGACATAACAGACTTCCCA
**qPCR**	*ABCA5*	ACAGCTGCTTATTTTGCCGTT	ACCTCCCTAATTGCAGTGGAC
**qPCR**	*SLC11A2*	CTGGCTCCCGGAATATGGAG	GGTGGATACCTGAGTGGCTG
**qPCR**	*CLK1*	TTGTCCAGGACGATGAGACAC	ATTTGCAGCGCTTGTTCTCC
**qPCR**	*THOC1*	CCATTGAACAGGCAGACCCT	GCTGGAAGAAGTGAGGGCTT
**qPCR**	*ATM*	GCGTGGCTAACGGAGAAAAG	CACTGCACTCGGAAGGTCAA

Supplementary Table 2. Possible disease-causing variants in the filtered WES data

Gene	Chromosome	Variant	Zygosity	Consequence	CADD	PolyPhen	SIFT
GNAS	20	c.462C>G; p.Tyr154Ter	Het	Nonsense	36	Possible damaging	Deleterious
TFRC	3	c.64C>T; p.ARG22Trp	Hom	Missense	25.7	Possible damaging	Deleterious
PIK3CD	1	c.1550G>A; p.Arg517Gln	Het	Missense	22.9	Possible damaging	Deleterious
CALD1	7	c.349C>T; p.Arg117Trp	Hom	Missense	26.9	Possible damaging	Deleterious

WES: Whole exome sequencing

Supplementary references

1. Kechin A, Boyarskikh U, Kel A, Filipenko M. cutPrimers: A New Tool for Accurate Cutting of Primers from Reads of Targeted Next Generation Sequencing. J Comput Biol. 2017;24(11):1138-43.

2. Dobin A, Davis CA, Schlesinger F, Drenkow J, Zaleski C, Jha S, et al. STAR: ultrafast universal RNA-seq aligner. Bioinformatics. 2013;29(1):15-21.

3. Robinson MD, McCarthy DJ, Smyth GK. edgeR: a Bioconductor package for differential expression analysis of digital gene expression data. Bioinformatics. 2010;26(1):139-40.

Supplementary figure legends

Supplementary Fig. 1. Gating strategy of flow cytometric surface and intracellular staining

Supplementary Fig. 2. A. Representative pictures of failure to thrive and facial dysmorphism in the patient. **B**. Brain MRI image

Supplementary Fig. 3. A. Top clonal proportions of the T and B cell repertoire. The graphs show the occupation of top 100 shared clones in total repertoire. **B**. Frequency of hydrophobicity of amino acids at position 6 and 7 of TRB CDR3 sequences. **C**. Relative mRNA expressions of several genes selected from transcriptome data.

Supplementary Fig. 4. The summary of TfR1 deficiency in a patient with combined immunodeficiency

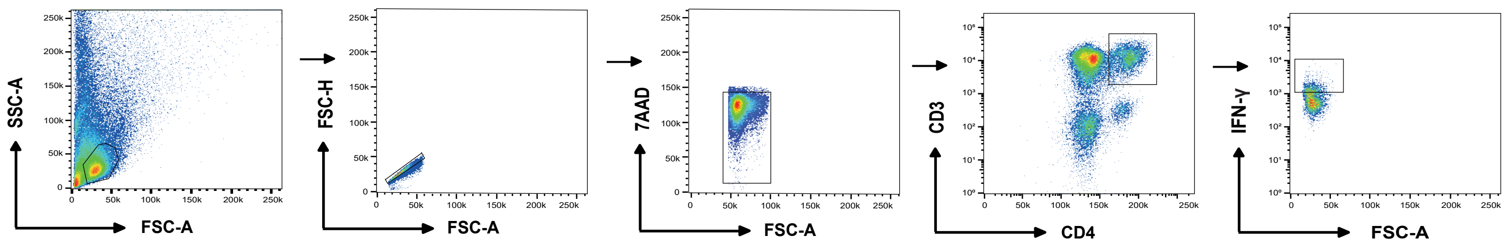

Supplementary Fig. 1

A

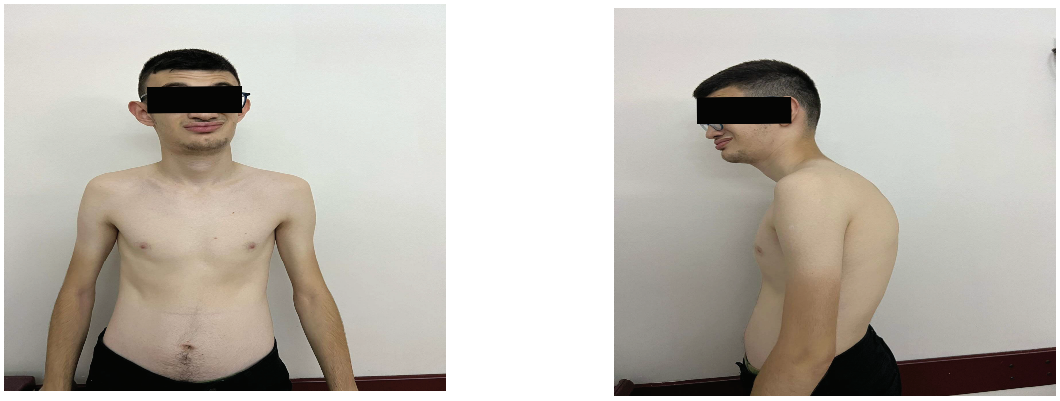

B

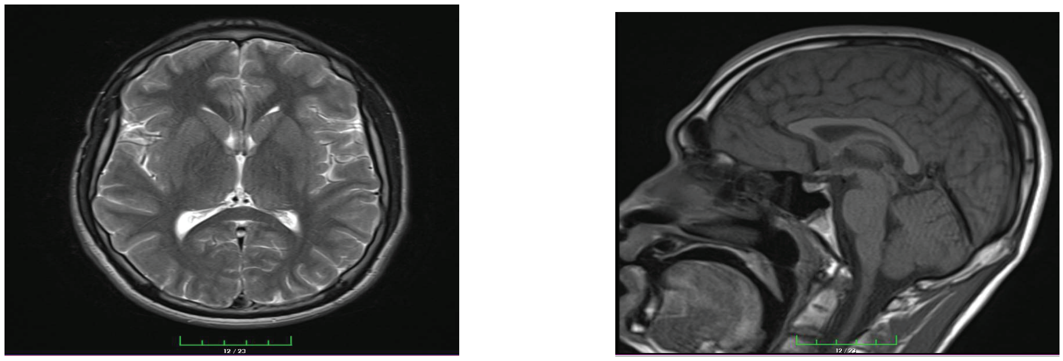

Supplementary Fig. 2

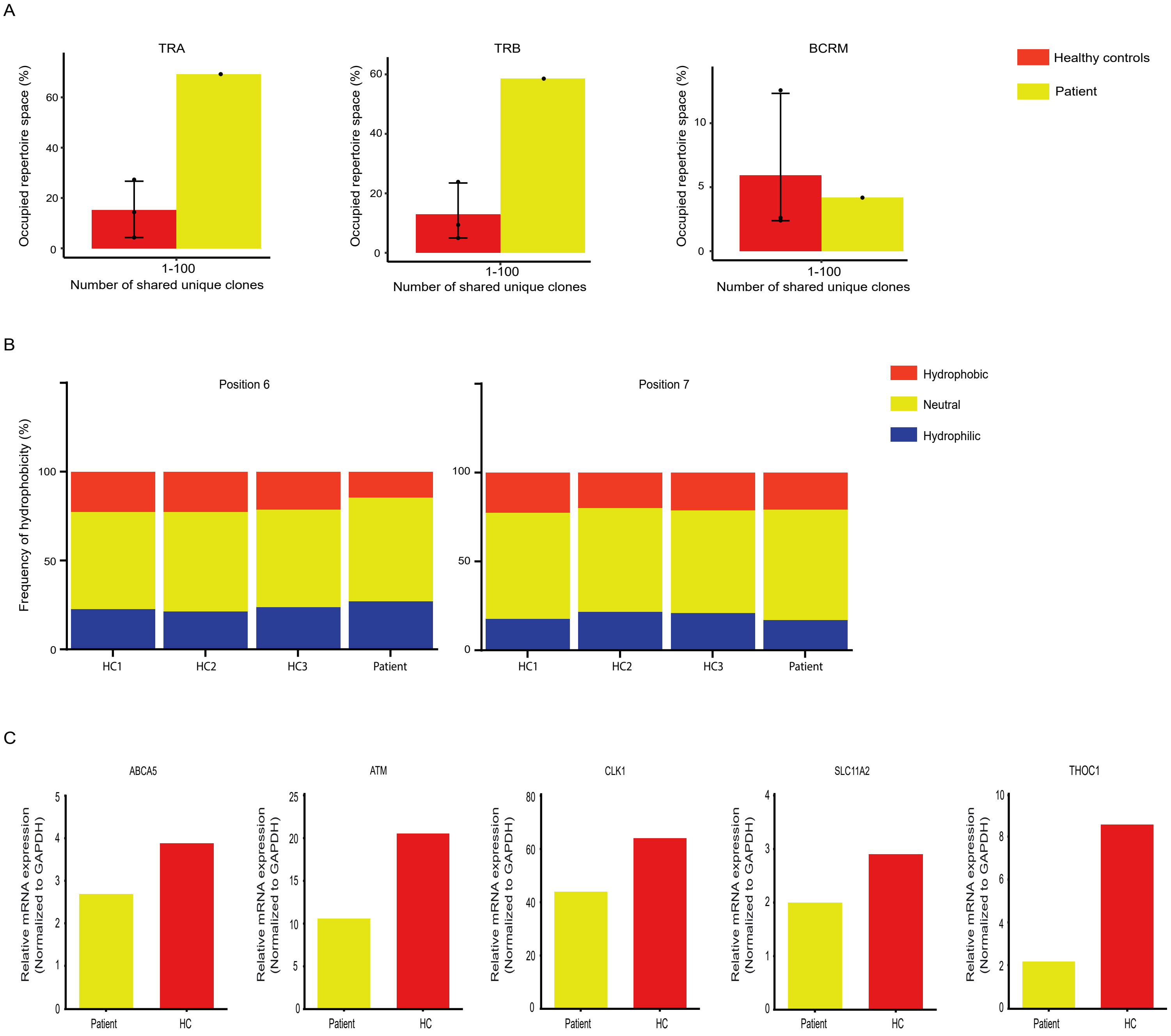

Supplementary Fig. 3

A novel homozygous germline mutation in transferrin receptor 1 (TfR1) leads to combined immunodeficiency and provides new insights into iron-immunity axis

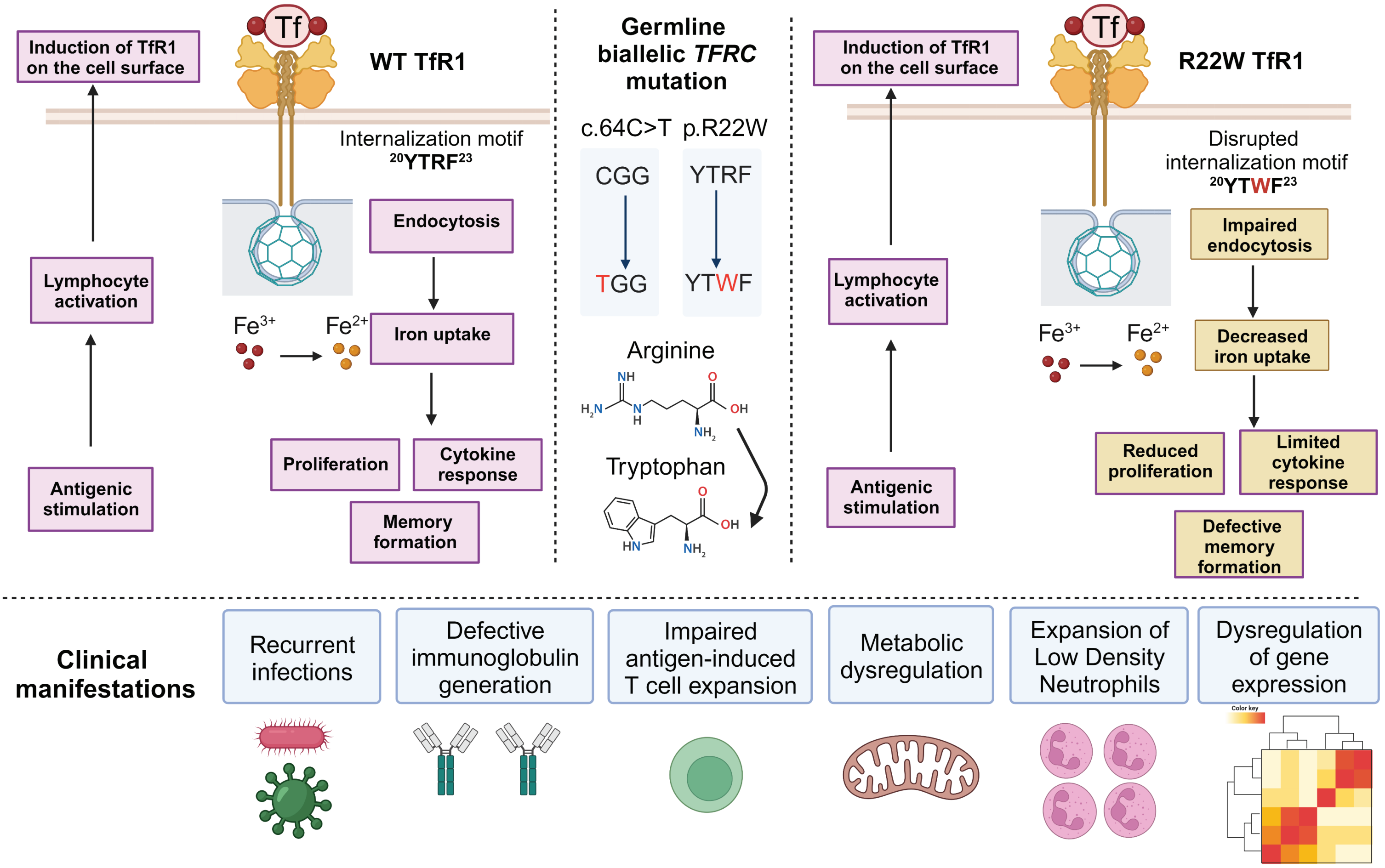

Supplementary Fig. 4
